# Trends in Antibiotic Treatment of Acute Otitis Media and Treatment Failure in Children, 2000–2011

**DOI:** 10.1371/journal.pone.0081210

**Published:** 2013-12-04

**Authors:** Leah J. McGrath, Sylvia Becker-Dreps, Virginia Pate, M. Alan Brookhart

**Affiliations:** 1 Department of Epidemiology, University of North Carolina, Chapel Hill, North Carolina, United States of America; 2 Department of Family Medicine, University of North Carolina, Chapel Hill, North Carolina, United States of America; Fondazione IRCCS Ca' Granda Ospedale Maggiore Policlinico, Università degli Studi di Milano, Italy

## Abstract

**Objectives:**

Guidelines to treat acute otitis media (AOM) were published in 2004. Initial declines in prescribing were shown, but it's unknown if they were sustained. We examine trends in antibiotic dispensing patterns to treat AOM among a large population of children. We also document trends in antibiotic failure.

**Study Design:**

Children aged 3 months to 12 years with an AOM diagnosis, enrolled in a commercial claims database between January 1, 2000-December 31, 2011 were included. Pharmacy claims within 7 days of diagnosis were searched for antibiotic prescriptions. Antibiotic failure was defined as a dispensing of a different antibiotic class within 2-18 days after the first prescription. We analyzed trends in antibiotic use and failure by class of antibiotic and year.

**Results:**

We identified over 4 million children under 13 years with AOM. The proportion of antibiotic dispensing decreased from 66.0% in 2005 to 51.9% in 2007, after which the instances of dispensing rebounded to pre-guideline levels. However, levels began decreasing again in 2010 and the antibiotic use rate in 2011 was 57.6%. Cephalosporin prescriptions increased by 41.5% over eleven years. Antibiotic failure decreased slightly, and macrolides had the lowest proportion of failures, while all other classes had failure rates around 10%.

**Conclusions:**

In recent years, antibiotic dispensing to treat AOM remains high. In addition, the use of broad-spectrum antibiotics is increasing despite having a high rate of treatment failure. Overprescribing of antibiotics and use of non-penicillin therapy for AOM treatment could lead to the development of antibiotic-resistant infections.

## Introduction

In children, acute otitis media (AOM) is one of the most common infections for which antibiotics are prescribed.[1]_ENREF_1 The overuse of all types of antibiotics, as well as using broad-spectrum antibiotics can lead to antibiotic-resistant bacteria[2] and higher medical costs. Initiatives have been implemented to increase the appropriate use of antibiotic prescribing among physicians._ENREF_2 In 2004, the American Academy of Pediatrics (AAP) and the American Academy of Family Physicians (AAFP) released guidelines for management of AOM in children <13 years that encouraged using antibiotics less frequently. These guidelines include observation without treatment as an option for certain age groups with non-severe or uncertain cases of AOM, and the use of high-dose amoxicillin as first-line treatment for severe cases or in younger age groups.[3] In 2013, these guidelines were updated to include more stringent diagnosis criteria and more specific definitions of categories for which antibiotics were recommended.[4]

Because of the detrimental effects of over-prescribing antibiotics, there has been a considerable effort to understand how the use of these drugs has changed over time. Several studies in the late 1990s showed that overall antibiotic prescribing was decreasing even prior to the AAP/AAFP guidelines;[5], [6] however, some found that use of broad-spectrum antibiotics was increasing.[7], [8] A study using national survey data from 2002 to 2006 found that immediately after the initial guidelines were published there was a non-significant decrease in encounters where antibiotics were prescribed for AOM, and significant increases in the use of amoxicillin as well as increases in the use of cephalosporins.[9] In addition, treatment failure is an important outcome because further prescribing of antibiotics, including broad-spectrum antibiotics, is often the result. Several studies have included clinical treatment failure as an endpoint, but these were small[10], [11] or focused solely on one type of antibiotic.[12], [13] One meta-analysis did report a higher risk of clinical failure for macrolides compared to amoxicillin, however only included studies through September 2008.[14] Relatively little is known about patterns of use of antibiotics to treat AOM and treatment failure during the last five years.

To address these gaps in knowledge, we sought to examine treatment patterns for AOM among a very large population of children with commercial insurance using contemporary data. We report proportions of dispensed antibiotic prescriptions to treat AOM five years before and seven years after the initial 2004 AAP/AAFP guidelines. We also examine trends in antibiotic failure over time and compare the risk of failure by type of initial prescription.

## Materials and Methods

### Study Population

We analyzed data from the MarketScan Commercial Claims and Encounters database (available for purchase from Truven Health Analytics) between January 1, 2000-December 31, 2011. The data capture patient-level data on inpatient, outpatient, and prescription drug claims from approximately 150 large employers and health plans that insure employees, early retirees and dependents. This database includes 23 million children over ten years. Drug information represents prescriptions that were collected at a pharmacy.

Children aged 3 months to 12 years were included in the study sample. Outpatient diagnoses are coded using *International Classification of Diseases, Ninth Revision, Clinical Modification* (*ICD-9-CM*) codes. We identified children with a diagnosis code for AOM as either the primary or secondary diagnosis by the following *ICD-9-CM* codes: 382.x, 384.2. These codes were chosen as the AAP/AAFP guidelines were specific to these conditions. We also compared our main cohort to children with a diagnosis of otitis media with effusion (OME) (codes 381.0-381.4) – while the guidelines were not focused on these conditions; we thought it useful to also characterize prescribing trends for children with an otitis media diagnosis where clinical guidelines suggest using antibiotics less often. Children had to be enrolled for at least 90 days without an otitis media diagnoses to be considered a new illness episode. We analyzed only the first new illness episode per child, to make sure we did not include recurrent disease. We further categorized children into age categories: <2 years, 2–3 years, and 4+ years as prescribing patterns could vary by age.

### Outcomes

For the initial otitis media diagnosis, we searched prescription drug claims for all types of oral antibiotics. A drug claim was considered linked to an outpatient diagnosis if it occurred within 7 days of the outpatient visit. We divided antibiotics into six categories: amoxicillin, amoxicillin/clavulanate, unspecified penicillins, macrolides, cephalosporins, and other antibiotics. If more than one antibiotic was filled during the week following the diagnosis, we counted each antibiotic in its respective class, but the encounter only counted once as a visit for which antibiotics were prescribed. Antibiotic failure was defined as children who filled a prescription for an oral antibiotic, or were given injectable or intravenous antibiotics (see Appendix S1 for codes) of a different class within 2–18 days after the first prescription. We further defined “early” and “late” failure to be 2–7 days and 8–18 days respectively. As a secondary analysis, we limited our failure results to children with no other diagnosis code of infection which could likely result in additional antibiotic prescriptions (*ICD-9-CM* codes: 003–006, 033–034, 036, 038, 320, 322, 466.0, 481–486, 599.0).

### Statistical Analysis

The proportion of children who were prescribed antibiotics was compared to the proportion of children who were not prescribed antibiotics. We evaluated overall antibiotic use and specific antibiotics by year and by age category. To assess trends that appeared approximately linear, we fit a linear regression to the data and report the p-value for the trend. Differences in proportions were assessed using a chi-square test. All analyses were performed using SAS 9.2 (SAS Institute, Cary, North Carolina).

### Ethics Statement

This study was approved by the institutional review board of University of North Carolina at Chapel Hill. As this study used de-identified data as a secondary data analysis and was classified as research involving no greater than minimal risk, the requirement of obtaining informed consent was waived.

## Results

There were 4,629,460 children aged 3 months – 12 years identified with an AOM diagnosis, after dropping 101 children who had a missing age. Overall, the mean age of the cohort was 3.8 years, while the median was 3.0 years. Children who were given antibiotics were of similar age and gender as those who were untreated (Table 1). There were differences in region in prescribing patterns – the west region had higher rates of prescribing antibiotics (66% vs 59%, 59%, and 60% for the south, northeast, north central, respectively). In our secondary cohort of children with an OME diagnosis, 42.2% received an antibiotic. These children were slightly older, but were similar in other demographic characteristics.

**Table 1:** Characteristics of children aged 3 months to 12 years with a diagnosis of otitis media, 2000–2011.

	Acute Otitis Media				Otitis Media with Effusion			
	Antibiotics		No Antibiotics		Antibiotics		No Antibiotics	
	N = 2,784,108		N = 1,845,352		N = 382,844		N = 523,618	
	N	%	N	%	N	%	N	%
Mean age	3.8	3.52	3.9	3.53	4.1	3.6	4.4	3.5
(Std), years								
Male sex	1,434,900	51.5	956,916	51.9	197,146	51.5	280,059	53.5
Region								
Northeast	350,074	12.6	244,862	13.3	49,556	12.9	84,644	16.2
North central	737,316	26.5	492,061	26.7	92,455	24.2	131,932	25.2
South	1,157,846	41.6	810,230	43.9	173,537	45.3	226,192	43.2
West	504,608	18.1	261,696	14.2	62,990	16.5	71,919	13.7
Unknown	34,264	1.2	36,503	2.0	4,306	1.1	8.931	1.7

**Figure 1:**
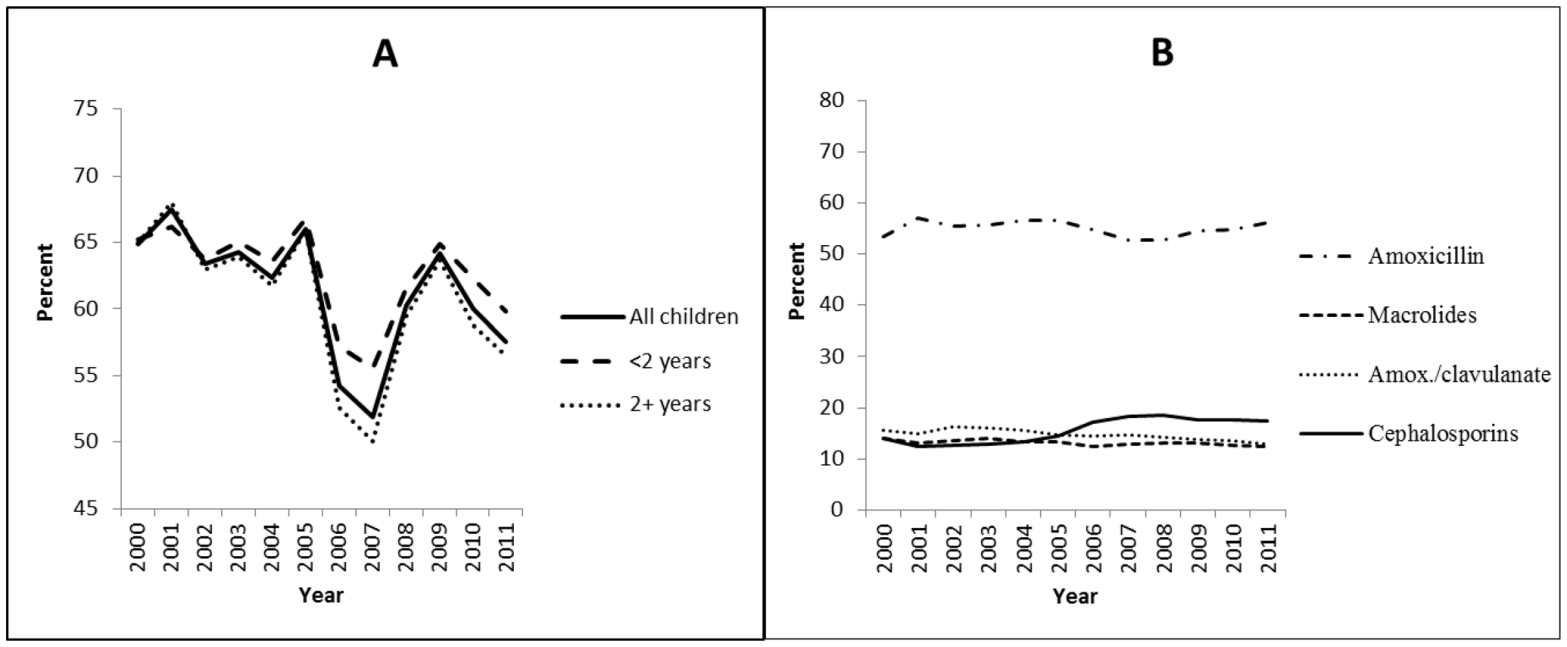
Initial dispensed antibiotics to treat acute otitis media among children, by age (Panel A) and antibiotic type (Panel B).

Over the 12 year period, 60.1% of children with AOM diagnoses were dispensed antibiotics. The proportion of dispensing decreased from 66.0% in 2005 to 51.9% in 2007, after which the instances of antibiotic dispensing rebounded to pre-AAP/AAFP guideline levels. However, levels began decreasing again in 2010 and antibiotic use in 2011 was 57.6%. Of all antibiotics dispensed, 54% were for amoxicillin. Use of amoxicillin decreased slightly – from 57% to 53% between 2005–2008, but increased to pre-guideline levels by 2011 (Figure 1A). This trend was seen in children of all ages, although children 2 years and older had slightly less antibiotic use.

Overall, non-amoxicillin containing antibiotics represented 30.6% of all first antibiotic dispensings. While macrolide use remained constant at ∼13% of dispensed antibiotic prescriptions and amoxicillin/clavulanate declined slightly (p = 0.002), cephalosporin use increased over time. Cephalosporin prescriptions increased from a low of 12.3% in 2001, peaked in 2007, and leveled out at 17.4% in 2011 (p = 0.0004). This was a 41.5% increase over eleven years. (Figure 1B). Cefdinir accounted for 70.8% of all cephalosporin dispensings, and 96% of cephalosporin dispensings were either second- or third-generation drugs.

**Figure 2:**
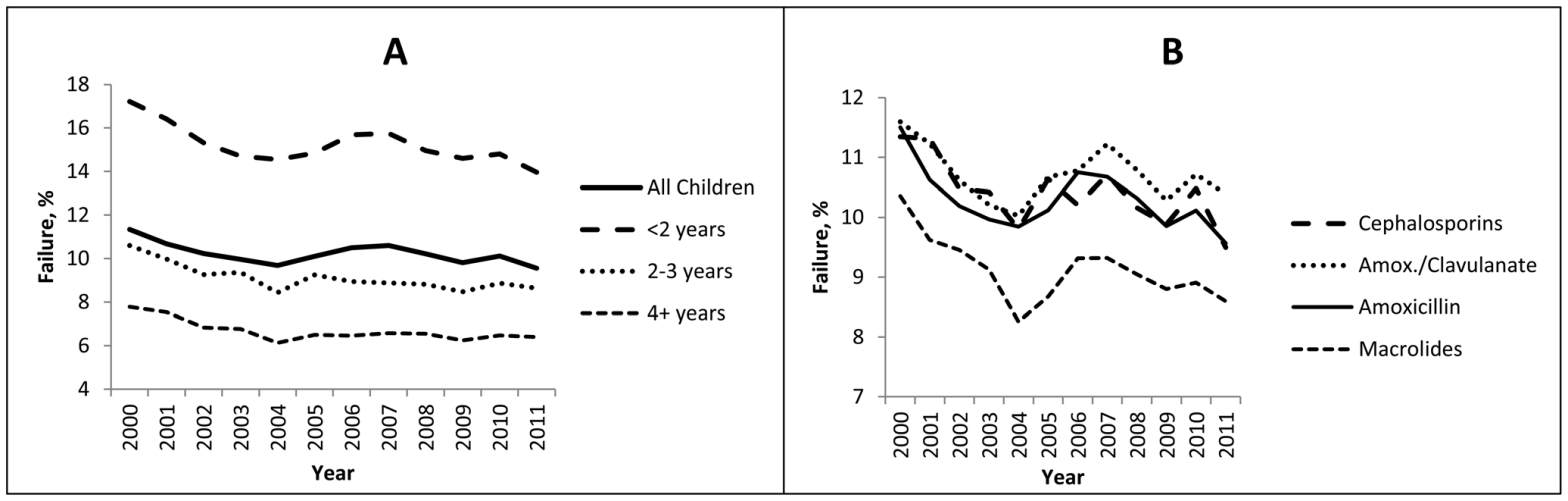
Antibiotic failure among children with an acute otitis media diagnosis by age categories (Panel A) and initial antibiotic type (Panel B).

Among children with AOM and with one initial prescription, 10.1% required an additional antibiotic within 2–18 days over the 12 year period (Table 2). Antibiotic failure decreased slightly over time (p = 0.03) and only 9.6% required a second prescription in 2011 (Figure 2). Children less than 2 years of age had the highest levels of failure (Figure 2A). Cephalosporins, amoxicillin/clavulanate and amoxicillin had similar failure proportions, while macrolides had a lower failure rate (p<0.0001) (Table 2); however, all antibiotic classes had a similar decline over time (Figure 2B). When children with diagnosis codes for other infections were excluded (N = 3,731), the failure proportions were similar (data not shown). Among children requiring only one antibiotic for the second dispensing, cephalosporins were the most common second prescription (37.7%) and the majority of children who received amoxicillin, amoxicillin/clavulanate and macrolides as first agents were switched to a cephalosporin (Table 3). For those started on a cephalosporin, the majority were switched to amoxicillin/clavulanate. Among children who experienced a treatment failure with one initial therapy, 6.4% received multiple (2 or more) antibiotics in subsequent dispensings.

**Table 2:** Antibiotic failures* among children with an acute otitis media diagnosis and 1 initial dispensed antibiotic.

	Overall		Early**		Late***	
	# Failures	Failure proportion, %	# Failures	Failure proportion, %	# Failures	Failure proportion, %
All antibiotics	279,102	10.1	94,925	3.4	184,177	6.6
Cephalosporin	44,485	10.2	14,074	3.2	30,411	7.0
Macrolide	30,627	8.9	14,085	4.1	16,542	4.8
Amox./clav.	40,769	10.6	13,766	3.6	27,003	7.0
Amoxicillin	159,806	10.1	51,695	3.3	108,111	6.8
Other	3,312	11.5	1,263	4.4	2,049	7.1

* Failure is defined as having an additional, different class of antibiotic prescription within 2–18 days of the index prescription.

** Early failure = 2–7 days.

*** Late failure = 8–18 days.

**Table 3:** Class of second antibiotic prescription by type of first antibiotic prescription, among children with an acute otitis media diagnosis, 1 initial dispensed antibiotic, and 1 additional dispensed antibiotic.

1^st^ Prescription		2^nd^ Prescription (%)				
	N	Amoxicillin	Amox./clav.	Macrolide	Cephalosporin	Other
Amoxicillin	149,110	–	31.7	22.8	41.5	3.9
Amox./clav.	38,343	8.9	–	26.7	57.6	6.6
Macrolide	28,833	17.4	28.4	–	46.5	7.4
Cephalosporin	41,842	10.6	44.0	37.3	–	7.9
Other	3,078	16.2	21.5	26.5	35.5	–

## Discussion

In this large, commercially-insured population of children following the initial 2004 AAP/AAFP guidelines, we observed an initial decrease in encounters with dispensed antibiotics to a low of 51.9%. These estimates are lower than a study limited to two years after the implementation of the guidelines, which estimated that 84% of children were given prescriptions.[9] The discrepancy may lie in the fact that Coco et al. used prescribed antibiotics while our study used dispensed antibiotics. Two studies have reported that approximately 18–25% of people do not fill antibiotic prescriptions.[15], [16] Furthermore, using dispensed antibiotics captures the “observation + safety-net” option, where physicians can write a prescription that the parent can fill if the child shows no improvement after a specific length of time. Using dispensed prescriptions as the outcome may give a more accurate picture of the effect of the guidelines – as this observation period was one way for physicians to decrease antibiotic use. One study estimated in 2006, that surveyed physicians used the observation option in 15% of their patients.[17]

We observed that the decline in dispensing following the implementation of the initial guidelines was not sustained. Although most recent data suggests that dispensing is again on the decline, antibiotic use has been near pre-guideline levels in years after 2007. This could indicate that physicians may have returned to past prescribing patterns. This agrees with a survey of physicians that reported increasing discordancy with the guidelines in choice of prescribed antibiotics over time.[17] Explanations for this trend may be that the initial awareness of the guidelines may have worn off, physicians have become overloaded with clinical practice guidelines[18] or that physicians are generally more likely to prescribe inappropriate antibiotics over time.[19] Physicians might also have recognized that patients may have better outcomes if given antibiotics. Recent randomized controlled trials have shown that children given amoxicillin[20] or amoxicillin-clavunate[21] had reduction in clinical failures as compared to placebo. It is interesting to note that 42.2% of children with a diagnosis of OME were treated. Clinical treatment guidelines for OME recommend watchful waiting for 3 months for otherwise healthy children, and include a specific recommendation to avoid antimicrobial use.[22] This may be a population that could be targeted for reduced antibiotic use. In fact, the 2013 update to the AOM recommendations encourages physicians to employ stricter diagnosis criteria to differentiate AOM and OME, and limit subsequent prescribing for uncertain AOM diagnoses.

Additionally, we showed an increasing use in cephalosporins used as initial treatment for AOM. Other studies have shown similar increases for non-first-line antibiotics, particularly with the antibiotic cefdinir.[7], [9] Although cephalosporins may be used in the case of penicillin allergy, it is estimated that the prevalence of allergy is <10%.[23] It is likely that increased use of cephalosporins may be due to an easier dosing schedule or higher tolerability[24] including fewer instances of diarrhea – a side effect that is commonly seen with amoxicillin/clavulanate.

An encouraging finding is that treatment failures have decreased, even with an increase in overall treatment since the initial guidelines were published. Our estimates are similar to the decreasing trend reported in a large, observational study of children in Boston treated with high-dose amoxicillin, where the relapse rate was 8.9% in 2004.[13] One explanation for decreasing failure rates could be due to lower prevalence of antibiotic-resistant strains of *Streptococcus pneumoniae* since the introduction of the pneumococcal conjugate vaccine (PCV). PCV-7 was introduced in 2001,[25] although supply issues limited availability until 2004. In pre-vaccine years, *Streptococcus pneumoniae* was a leading cause of AOM[26] and some strains have been shown to be resistant to certain antibiotics. Since vaccine introduction a small decline in AOM has been documented.[27] Additionally, PCV-13 was introduced in 2010, and covers a common strain that has been associated with antibiotic resistance (serotype 19A).[28], [29] The lower levels of treatment initiation and failure in the last year of our study may be due to the impact of PCV-13 on lowering the overall prevalence of otitis media caused by *S. pneumonia*, as well as decreasing antibiotic resistant strains.

We also show that macrolides had a lower failure rate, while cephalosporins, amoxicillin, and amoxicillin/clavulanate had similar, higher rates of failure. Interestingly, macrolides had a higher failure rate in the early failure window (3–7) days, but had a lower failure rate in the late failure window. Possible explanations for the lower overall rates of failure seen among macrolides could be that physicians are using these drugs to treat to viral infections, which would have resolved regardless of treatment, or that the shorter course (5 days) results in higher compliance and better outcomes. Recent studies have reported contradictory results when comparing failure rates between classes of antibiotics. One population based, observational study reported similar positive findings for macrolides as our study- azithromycin had a 12% lower odds of failure as compared to amoxicillin._ENREF_22[30] However, a meta-analysis suggested that macrolides had a higher rate of clinical failure as compared to amoxicillin-containing regimens (risk ratio 1.31 (95% CI: 1.07–1.60). The discrepancy may lie with the varying definitions of treatment failure. We found little consistency in the literature with regards to what events were considered failures and the length of time that was considered for the failure window. _ENREF_11 Therefore, it makes it difficult to compare failure of different types of antibiotics across studies. Though our results suggest a slightly lower failure rate among macrolides, these data should be interpreted with caution when considering a change in prescribing patterns, as we could not incorporate antibiotic susceptibility testing of tympanocentesis cultures. While the overall failure rate in our study decreased over the 12 year period, possibly due to the introduction of PCV, it is worrisome that the increasing use of cephalosporins as the initial treatment could result in the use of more antibiotics overall.

There were several limitations to this study. First, we were unable to distinguish the cause of the changing trends in treatment patterns. It is possible that treatment patterns did actually change in response to the guidelines. However, it is also possible that the diagnosis of acute otitis media changed over time – if physicians were diagnosing milder cases, this could create an appearance of a decreased treatment effect. Therefore, it is possible that the decrease in overall dispensing we showed between 2005 and 2007 was due to a change in diagnosing, as opposed to a change in treatment patterns. Second, we did not have information on weight in the dataset. This prohibited the assessment of whether a certain antibiotic was classified as “high-dose,” particularly amoxicillin containing antibiotics. Third, we did not take into consideration if a child had either a past failure or an allergy to penicillin, which could influence what type of initial antibiotic was prescribed. Fourth, as the study population was limited to commercially insured children, it may be difficult to generalize to either noninsured or Medicaid populations. Further work should be done to characterize the patterns of antibiotic use in these populations. Fifth, our study was limited to dispensed prescriptions where a claim was filed to the insurance company. Antibiotics given as a free sample from a physician's office or through a pharmacy program giving free courses of generic antibiotics would not be captured, and thus we may have underestimated the actual amount of antibiotic use. However, it has been estimated that only 5% of children received free samples in 2004,[31] and therefore we believe that the misclassification in our estimates would be minimal. Finally, our definition of antibiotic failure does not capture whether the failure was truly a clinical failure, a response to an adverse reaction to the first drug, or due to subsequent infections. However, restricting the population to those episodes where AOM was the only infectious diagnosis code should help to more validly estimate treatment failure. Our measure of failure does show the overall need for multiple prescriptions regardless of the underlying mechanism.

Following the release of the initial AAP/AAFP guidelines, the practice of antibiotic dispensing appears to have initially decreased, followed by a rebound to pre-guideline levels. While the most recent estimates indicate antibiotic treatment may again be declining, overall use of antibiotics remains high. Additionally, we document an increasing use of cephalosporins for first-line treatment of AOM. This is discouraging in light of higher rates of treatment failure observed with cephalosporins. Overprescribing of antibiotics and use of non-amoxicillin therapy for AOM treatment are worrisome as these deviations from treatment guidelines could lead to the development and transmission of antibiotic-resistant infections.

## Supporting Information

Appendix S1 **Codes for intravenous antibiotics included in the failure analysis.**
(DOCX)
