# Supplementary material for: Trends in Antibiotic Treatment of Acute Otitis Media and Treatment Failure in Children, 2000–2011
Source: PLoS One. 2013 Dec 4;8(12):e81210. doi: 10.1371/journal.pone.0081210 (PMC3852237; doi:10.1371/journal.pone.0081210)
Supplement: Appendix S1 — Codes for intravenous antibiotics included in the failure analysis. (DOCX) [file pone.0081210.s001.docx]

| CPT Code | Definition |
| --- | --- |
| J0690 | Cefazolin |
| J0713 | Ceftazidime |
| J0692 | Cefepime |
| J0696 | Ceftriaxone |
| J3370 | Vancomycin |
| J1580 | Garamycin gentamicin |
| J3260 | Tobramycin |
| J0278 | Amikacin Sulfate |
| J1840 | Kanamycin |
| J1956 | Levofloxacin |
